# A Multi-Task Learning Model Based on DTP-MMoE for Identification of Olive Oil Multi-Adulteration Using Raman Spectroscopy

**DOI:** 10.3390/foods15112030

**Published:** 2026-06-05

**Authors:** Xuewen Qin, Yulong Chen, Bing Li, Shan Zeng, Gaoxiang Mei, Chen Yu

**Affiliations:** 1 School of Mathematics and Computer Science, Wuhan Polytechnic University, Wuhan 430023, China; 2 College of Medicine and Health Science, Wuhan Polytechnic University, Wuhan 430023, China

**Keywords:** olive oil adulteration, Raman spectroscopy, multi-task learning, non-destructive detection, food authenticity

## Abstract

Olive oil adulteration with low-cost vegetable oils poses a serious food safety concern. This study proposes a Dynamic Task Priority Multi-Gate Mixture-of-Experts (DTP-MMoE) model based on Raman spectroscopy to simultaneously perform the qualitative discrimination of adulteration types and quantitative prediction of adulteration ratios. The model learns shared spectral representations through expert networks and task-specific gating mechanisms, while a dynamic task priority loss function adaptively balances optimization between the classification and regression tasks. Experimental results demonstrated that the DTP-MMoE model achieved a classification accuracy of 99.15% and a coefficient of determination (R^2^) of 0.99 for prediction, significantly outperforming conventional single-task and multi-task baselines. Ablation studies confirmed the critical contributions of the gating mechanism, expert network configuration, and dynamic weighting strategy. Furthermore, external validation on commercial blended oil samples not involved in training yielded a mean absolute error (MAE) of 0.317%, an RMSE of 0.459%, and a MAPE of 6.34%, demonstrating good generalization capability. The proposed method provides an efficient, non-destructive, and reliable analytical tool for rapid screening of olive oil authenticity, showing considerable promise for application in food quality control and regulatory practice.

## 1. Introduction

Olive oil is highly valued worldwide for its rich nutritional profile and health-promoting properties. It contains abundant natural bioactive compounds, including unsaturated fatty acids, polyphenols, and carotenoids. Long-term consumption of olive oil may contribute to the prevention of chronic diseases such as cardiovascular disease, atherosclerosis, and hypertension; consequently, it is widely regarded as an important component of a healthy dietary pattern [1]. However, owing to its distinctive sensory attributes and relatively high production costs, olive oil is priced substantially higher than common vegetable oils such as rapeseed oil, sunflower oil, and corn oil. This price disparity has encouraged economically motivated adulteration, particularly the illegal addition of low-cost vegetable oils to olive oil [2]. Such practices not only seriously compromise the nutritional properties and flavor integrity of olive oil but also disrupt market order and infringe upon consumer rights, causing olive oil adulteration to gradually evolve into an increasingly serious food safety issue. According to statistics, the number of reported adulteration cases has increased by as much as 87.5% over the past decade [3]. Therefore, developing rapid, accurate, and non-destructive techniques for adulteration detection is of great significance for ensuring food safety, maintaining fair trade, and preserving consumer trust.

Among conventional analytical methods, gas chromatography (GC) and liquid chromatography (LC) are regarded as authoritative approaches for adulteration identification because of their high sensitivity and selectivity [4]. These methods can accurately identify and quantify characteristic components in oil samples, such as fatty acids, sterols, and polyphenolic compounds. Nevertheless, they generally require complex sample pretreatment, are time-consuming, and are destructive, making them unsuitable for rapid on-site screening. Accordingly, spectroscopic techniques such as Raman spectroscopy, Fourier-transform infrared spectroscopy (FT-IR), near-infrared spectroscopy (NIR), and fluorescence spectroscopy have gradually become research hotspots due to their rapid, non-destructive, and easy-to-operate characteristics, and they have demonstrated considerable application potential in olive oil authenticity assessment [5]. Among these techniques, Raman spectroscopy offers unique advantages for the non-destructive detection of edible oil systems because of its sharp spectral peaks, strong molecular specificity, and low sensitivity to water interference, thereby providing an efficient solution for the rapid screening of adulteration. Continuous improvements in Raman instrumentation have greatly facilitated the on-site detection of edible oil adulteration, such as portable SORS analyzers capable of through-package olive oil quantification [6], compact fiber-optic Raman systems for flaxseed oil authentication [7], and miniaturized Raman devices coupled with durable SERS substrates for oil verification [8]. These advances underscore a clear trajectory toward lighter, more user-friendly Raman platforms for field-deployable edible oil quality control.

Chemometric and machine learning methods have been widely applied to the detection of olive oil adulteration, mainly focusing on two tasks. The first is the identification of adulteration categories using methods such as support vector machines (SVMs) and partial least squares discriminant analysis (PLS-DA) [9]. The second is the establishment of quantitative relationships between spectral features and blending ratios using methods such as partial least squares regression (PLSR) and support vector regression (SVR), thereby enabling the prediction of olive oil content. Machine learning models such as random forest (RF) and XGBoost have shown good performance in both classification and regression tasks owing to their strong nonlinear fitting capabilities [10]. When dealing with more complex data structures, deep learning algorithms represented by convolutional neural networks (CNNs), recurrent neural networks (RNNs) [5], long short-term memory networks (LSTMs) [11], and Transformers can achieve higher-accuracy identification and prediction through end-to-end feature extraction, showing notable advantages in the authentication of adulterated edible oils [12].

Previous studies have mostly adopted a single-task learning paradigm, in which separate models are constructed for adulteration type identification and content prediction. However, this stepwise strategy has inherent limitations. Although the two types of models are based on the same spectral data, their independent training processes fail to fully exploit and utilize the shared underlying chemical and spectral features between tasks, resulting in inefficient feature utilization. In addition, independent modeling workflows are complex and increase the costs of practical deployment and maintenance. More importantly, stepwise prediction may lead to inconsistent results. For example, a classification model may determine that a sample is adulterated, whereas the quantitative model may produce an adulteration ratio with low confidence. Such inconsistency can reduce the reliability of detection results and hinder regulatory decision-making. In fact, adulteration type identification and content prediction are closely related tasks. The simultaneous completion of qualitative discrimination and quantitative analysis is of great importance for practical regulation and quality control. Therefore, developing an integrated analytical method capable of performing both tasks simultaneously is expected to improve overall model performance, result consistency, and computational efficiency through feature sharing and joint optimization.

To address these issues, this study proposes a Raman spectroscopy-based Dynamic Task Priority Multi-Gate Mixture-of-Experts (DTP-MMoE) model, aiming to simultaneously and collaboratively accomplish qualitative discrimination of adulteration categories and quantitative analysis of adulteration ratios within a single analysis using one unified model. The proposed model learns shared spectral representations through a set of shared expert networks and performs adaptive feature fusion via task-specific gating mechanisms, thereby effectively capturing the relationships between the two tasks and improving the performance of each. Furthermore, a dynamic task priority loss function is designed to adaptively adjust optimization weights according to the learning difficulty of each task, promoting balanced optimization between qualitative discrimination and quantitative prediction. Systematic ablation experiments and comparisons with multiple benchmark methods demonstrate that DTP-MMoE performs excellently in both qualitative and quantitative analyses. Its integrated synchronous detection framework maintains high accuracy while offering greater efficiency and result consistency, providing an efficient and reliable analytical tool for the rapid screening of olive oil authenticity and showing clear application prospects in food quality and safety control as well as regulatory practice.

## 2. Materials and Methods

### 2.1. Research Workflow and Overall Design

**Figure 1:**
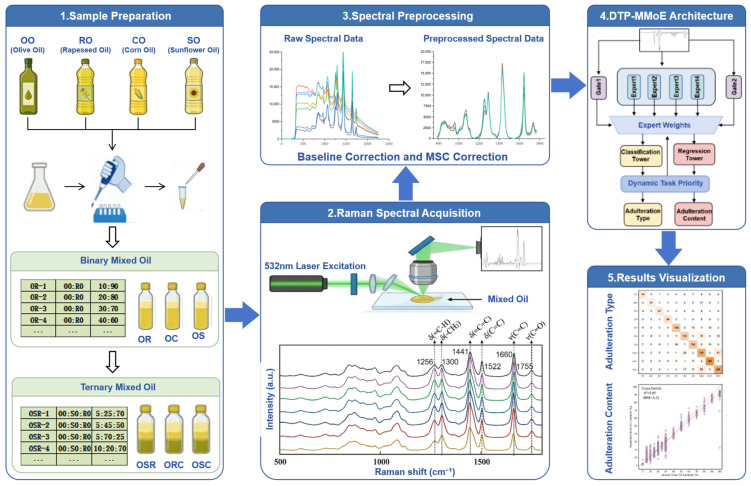
Flowchart of olive oil adulteration detection using a multi-task learning model based on DTP-MMoE.

The overall research framework of this study is shown in Figure 1. First, Raman spectral data from different blended samples were collected and preprocessed. Subsequently, a DTP-MMoE multi-task learning model was constructed to simultaneously perform blending type classification and regression-based prediction of olive oil content. Finally, the model performance and internal mechanisms were analyzed based on the experimental results.

### 2.2. Sample Preparation

This study used commercially available extra virgin olive oil and three common vegetable oils—sunflower oil (SO), rapeseed oil (RO), and corn oil (CO)—as research materials. To simulate real-world adulteration scenarios, two mixed-oil systems were prepared. First, in the binary mixture system, olive oil was separately blended with each of the other three oils, with the volume percentage of olive oil set from 10% to 90% at 10% intervals, resulting in nine concentration gradients. Second, in the ternary mixture system, the volume percentage of olive oil was set from 5% to 30% at 5% intervals, with specific formulations provided in Table S1. Three parallel samples were prepared for each mixing ratio to evaluate experimental reproducibility.

Sample preparation was conducted under constant-temperature conditions of 25 ± 2 °C. First, predetermined volumes of each pure oil were transferred into clean centrifuge tubes using a precision pipette. The samples were then treated in an ultrasonic processor (DL-480E, Shanghai Wuxiang Instrument Co., Ltd., Shanghai, China) for 10 min to ensure homogeneous mixing. Immediately after mixing, 1 mL of each blended oil sample was transferred into a quartz tube for Raman spectral acquisition. To minimize operational error, three different positions were selected for each parallel sample, and 10 repeated measurements were performed at each position.

### 2.3. Data Acquisition

Raman spectral data were collected using a benchtop MR-3CHANNELS micro-Raman spectrometer (Shanghai Oceanhood Opto-Electronics Tech Co., Ltd., Shanghai, China). A 532 nm continuous-wave laser was used as the excitation source, with a power of 40 mW and a linewidth of less than 0.2 nm [13]. The laser was focused onto the surface of the oil droplet on a glass slide through a 10× objective lens, and optimal focusing was achieved by adjusting the sample stage to enhance the Raman signal. The spectral acquisition parameters were set as follows: integration time, 1s; number of accumulations, 3. The collected spectral range covered 200–3600 cm^−1^, with a spectral resolution of 16 cm^−1^.

### 2.4. Raman Spectral Data Preprocessing

To improve spectral quality and reduce measurement-related interference, the raw Raman spectra were sequentially subjected to baseline correction, smoothing-based denoising, and scatter correction. First, baseline drift was removed using the built-in function of the instrument. Subsequently, the Savitzky–Golay filtering method, implemented using the SciPy library with a window length of 7 and a polynomial order of 3, was applied to suppress high-frequency random noise while preserving the characteristic spectral peak profiles. Finally, to eliminate the effects of light scattering caused by differences in the physical states of the samples, multiplicative scatter correction was performed using custom Python code. Specifically, the mean spectrum of all samples was used as the reference, each spectrum was fitted by linear regression, and spectral shape standardization was achieved through the corresponding correction coefficients.

### 2.5. Multi-Task Model Design

#### 2.5.1. MMoE Framework Module Design

A neural network with a multi-task structure can simultaneously predict multiple attributes within a single network framework [14]. In multi-task learning, various parameter-sharing mechanisms have been extensively investigated, including hard sharing, soft sharing, and hierarchical sharing. However, hard parameter sharing, in which all tasks share the same set of parameters, has obvious limitations. The objectives of different tasks may conflict with one another; for example, the gradient directions of classification and regression tasks may be inconsistent. This makes it difficult for the model to balance the requirements of different tasks and may even lead to “task interference.” To address this issue, the gate-based mixture-of-experts (MoE) mechanism has emerged as a more advanced solution. By using dynamic gating networks to select task-adaptive combinations of experts for different tasks, MoE enables each task to utilize the shared expert layers in an optimal manner. This avoids the rigidity of hard sharing and significantly improves the flexibility and performance of multi-task learning [15]. On this basis, this study designed an MMoE+DTP model based on gating networks. Its core architecture consists of four components: expert layers, gating networks, tower networks, and a dynamic weight adjustment layer. The expert layers extract general features, the gating networks dynamically assign weights to experts for each task, the tower networks output task-specific results, and the dynamic weight adjustment layer balances task losses. This design maintains parameter efficiency while achieving task decoupling through the gating mechanism.

**Figure 2:**
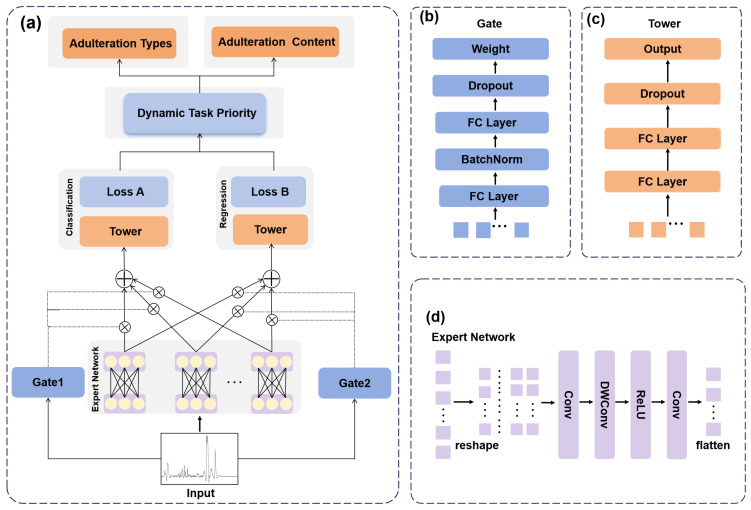
Architecture of DTP-MMoE. (**a**) Overall framework. (**b**) Gating network. (**c**) Tower network. (**d**) Expert network.

The expert networks are designed to automatically extract effective features from raw spectral data and provide fundamental representations for subsequent tasks. As shown in Figure 2b, after the input Raman spectral data are fed into the model, they are first transformed into two-dimensional features through a reshape operation. Local features are then extracted using convolutional layers (Conv) and depthwise separable convolutional layers (DWConv). The ReLU activation function is incorporated to enhance nonlinearity, and a feature vector is ultimately generated. Each expert is an independent feedforward network responsible for capturing feature patterns at different levels from the spectra.

The core function of the gating network is to generate “gating weights” for each task, dynamically select experts, and combine their outputs in a weighted manner, allowing different tasks to utilize the experts in differentiated ways [16]. Specifically, as shown in Figure 2c, each task—classification and regression—corresponds to an independent gating network, denoted as Gate1 and Gate2. The input to the gating network is the preprocessed spectral data. Each gating network contains two fully connected layers (FC layers), integrated with batch normalization to mitigate gradient vanishing and dropout to prevent overfitting. The output is normalized using a softmax function to generate a weight vector with the same length as the number of experts. This vector determines the contribution of each expert to the current task.

After receiving the expert outputs weighted by the gating network, the tower networks, shown in Figure 2d, generate the final prediction results through task-specific fully connected layers. The classification tower outputs the probability distribution of olive oil adulteration types through a softmax function after two FC layers, thereby performing multi-class classification. The regression tower directly outputs the continuous value of olive oil content after two FC layers, thereby performing regression prediction. This design allows different tasks to independently learn their optimal feature representations while maintaining parameter efficiency through shared expert layers and avoiding redundant computation.

#### 2.5.2. DTP Loss Function Design

In multi-task learning, the design of the loss function must balance the optimization requirements of different tasks and prevent a single task from dominating the training process. In this study, a dynamically weighted total loss function was adopted to improve the overall model performance by adaptively adjusting task weights, thereby overcoming the limitation of conventional fixed-weight strategies, which are difficult to adapt to dynamic changes among tasks.

To address task conflicts caused by differences in loss magnitude and convergence speed between the classification and regression tasks, this study introduced a dynamic task priority (DTP) mechanism. The total loss, $L_{\mathrm{total}}$, is obtained by the weighted summation of the losses of all tasks, as follows:

(1) $$L_{\mathrm{total}}\;=\;\sum_{t=1}^{T}\;w_{t}\left(t\right)\cdot L_{t}\;$$

where T denotes the number of tasks. In this study, T = 2, corresponding to the classification task for olive oil adulteration type identification and the regression task for olive oil content prediction. $L_{t}$ denotes the original loss of task *t*, such as the cross-entropy loss for the classification task and the mean absolute error for the regression task. $w_{t}$(*t*) represents the dynamic weight assigned to task t at training time step *t*.

The core idea of the dynamic weight $w_{t}$(t) is to allocate task weights according to the relative difficulty of each task, enabling the model to focus more on the task that is currently more difficult to optimize. It is calculated as follows:

(2) $${\;w}_{t}(t)\;=\;\alpha \cdot \sigma \left(\beta \cdot \frac{L_{\mathrm{smooth}}(L_{t})}{L_{\mathrm{smooth}}(L_{\mathrm{other}})}\right)$$

where σ denotes the sigmoid activation function, which constrains the weight to the interval (0, 1) and ensures the rationality of the assigned weight. α is a weight scaling factor that controls the overall magnitude of the dynamic weight, for example, to prevent a task from being ignored because of an excessively small weight. β is a weight sensitivity factor that amplifies differences in the loss ratio, making the weight more sensitive to changes in task difficulty; for instance, a task with a larger loss receives a higher weight. In this study, α was initially set to 1, and β was set to 2 but can be adjusted within the range of 0.5 to 5. $L_{\mathrm{smooth}}(L_{t})$ and $L_{\mathrm{smooth}}(L_{\mathrm{other}})$ denote the smoothed losses of task t and the other task, respectively, and are used to stabilize fluctuations in the original losses.

To reduce abrupt changes in task weights caused by batch-wise fluctuations in the original losses, an exponential moving average smoothing strategy was introduced, as follows:

(3) $$L_{\mathrm{smooth}}(L_{t})\;=\;\gamma \cdot L_{t}^{\mathrm{current}}+(1-\gamma )\cdot L_{t}^{\mathrm{history}}$$

where γ (set to 0.2) is the smoothing factor for the current loss, which balances the contributions of the current and historical losses. $L_{t}^{\mathrm{current}}$ denotes the original loss of task tin the current batch, whereas $L_{t}^{\mathrm{history}}$ denotes the historical smoothed loss. The historical smoothed loss is updated as follows:

(4) $$L_{t}^{\mathrm{history}}=\;\eta \cdot L_{t}^{\mathrm{current}}+(1-\eta )\cdot L_{t}^{\mathrm{history}}$$

where η (set to 0.2) is the historical loss update rate, which controls the updating speed of the historical loss.

### 2.6. Comparative Model Settings

To systematically validate the effectiveness of the proposed DTP-MMoE multi-task learning framework for olive oil adulteration detection, two types of comparative experiments were conducted: single-task learning models and multi-task learning models. All models were compared under the same data partitioning scheme and evaluation metrics to comprehensively assess the performance advantages of the proposed method in adulteration type identification and olive oil content prediction.

In the single-task learning comparisons, independent models were constructed for the classification and regression tasks, respectively. Both traditional machine learning methods and deep learning methods were selected for comparison, including support vector machine (SVM), random forest (RF), partial least squares (PLS), extreme gradient boosting (XGBoost), one-dimensional convolutional neural network (1D-CNN) [17], and long short-term memory network (LSTM). Specifically, SVM performs nonlinear classification and regression modeling through kernel functions and is suitable for high-dimensional small-sample data. RF improves model robustness and generalization ability by integrating multiple decision trees [18]. PLS, as a commonly used chemometric method in spectral analysis, can establish relationships between input variables and response variables while performing dimensionality reduction. XGBoost constructs high-performance ensemble models through a gradient boosting strategy and exhibits strong nonlinear fitting capability. 1D-CNN can directly extract local features from one-dimensional Raman spectra and capture variations in spectral peak neighborhoods. LSTM is well suited for modeling long-range dependencies in sequential data and can be used to explore continuous correlations among spectral variables. For SVM, RF, PLS, and XGBoost, models were constructed using two feature extraction methods, namely PCA and LDA. In contrast, 1D-CNN and LSTM directly used the preprocessed spectral sequences as input.

In the multi-task learning comparisons, to evaluate the effects of different parameter-sharing mechanisms and task-weight allocation strategies, hard parameter sharing, soft parameter sharing, multi-gate mixture-of-experts (MMoE), and uncertainty weighting were selected as baseline models. Hard sharing adopts the classical architecture of shared bottom representations and task-specific output layers, enabling parameter reuse; however, it is prone to task interference when task discrepancies are large. Soft sharing retains relatively independent parameter spaces for different tasks and promotes parameter information sharing through constraint mechanisms, thereby alleviating conflicts caused by hard sharing. MMoE shares multiple expert networks and assigns an independent gating network to each task, enabling selective sharing of task-relevant features. Uncertainty weighting dynamically adjusts the loss weight of each task at the loss-function level according to task uncertainty, thereby balancing the learning processes of different tasks. Building on these approaches [19], the proposed DTP-MMoE model further incorporates a dynamic task priority mechanism, jointly optimizing the classification and regression tasks from both feature-sharing and loss-weight adjustment perspectives.

### 2.7. Evaluation Metrics

To objectively evaluate the performance of each model in olive oil adulteration detection, five-fold cross-validation was adopted in this study. Specifically, all samples were randomly divided into five mutually exclusive subsets. In each iteration, four subsets were used as the training set, while the remaining subset was used as the validation set. This process was repeated five times, and the average results across all folds were taken as the final model performance, thereby improving the stability and reliability of the evaluation results.

For the classification task, namely olive oil adulteration type identification, accuracy, precision, and F1-score were used as evaluation metrics. Accuracy measures the proportion of correctly classified samples among all samples and directly reflects the overall discriminative ability of the model. Precision represents the proportion of samples predicted by the model as a given class that actually belong to that class, and can be used to evaluate the reliability of the classification results for each category. F1-score jointly considers precision and recall, providing a more comprehensive assessment of the overall performance of the model in multi-class classification tasks, especially for evaluating the balance of class discrimination performance.

For the regression task, namely olive oil content prediction, mean absolute error (MAE) and the coefficient of determination (R^2^) were used as evaluation metrics. MAE reflects the average absolute error between the predicted and true values; a smaller MAE indicates lower prediction error. R^2^ measures the ability of the model to explain variations in the target variable, with values closer to 1 indicating better fitting performance. In addition, the ratio of performance to deviation (RPD) and the range error ratio (RER) were also adopted to further assess the model’s predictive ability. RPD is defined as the ratio of the standard deviation of the reference values to the root mean square error of prediction (RMSEP). A higher RPD value indicates better model performance relative to the data variability. RER is defined as the ratio of the range of the reference values to RMSEP, with a larger RER reflecting stronger predictive capacity. These four metrics evaluate the quantitative prediction capability of the model from the perspectives of error magnitude and goodness of fit, respectively.

The software used in the experiments, such as Python 3.11.7 and PyTorch 2.8.1, as well as the environmental configurations, including the operating system and hardware parameters, are described in detail in Table S2 of the Supplementary Materials to ensure experimental reproducibility.

## 3. Results and Discussion

### 3.1. Spectral Feature Analysis

Raman spectra of edible oil samples with different blending types and blending ratios were analyzed in the range of 800–1800 cm^−1^. Overall, the spectra of different samples showed high consistency in peak position distribution, with the main characteristic peaks appearing at typical positions of 1156, 1265, 1300, 1441, 1526 and 1658 cm^−1^. This is because olive oil and other vegetable oils have similar chemical compositions, with fatty acid glycerides as their major constituents; therefore, the corresponding vibrational modes of their functional groups are highly consistent.

Although the peak positions of different samples were generally consistent, significant differences were observed in the intensities and relative variation trends of the characteristic peaks. These differences mainly arise from variations in the proportions of unsaturated fatty acids and other components among different oils. Therefore, the information contained in the Raman spectra is primarily reflected in changes in peak intensity rather than shifts in peak position, providing an important basis for subsequent classification and quantitative analysis.

**Figure 3:**
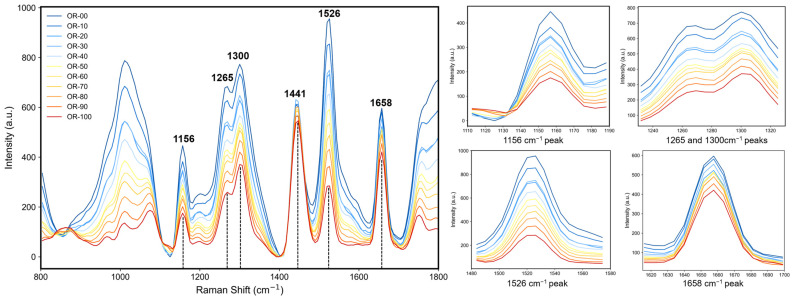
Raman spectra of representative binary adulterated oils in the 800–1800 cm^−1^ range. The magnified area on the right displays the five main characteristic peaks.

As shown in Figure 3, among all characteristic peaks, the peak located near 1656 cm^−1^ corresponds to the stretching vibration of C=C bonds in unsaturated fatty acids, and this vibrational mode is of great significance in the Raman spectroscopic analysis of edible oils. Previous studies have shown that olive oil is rich in monounsaturated fatty acids, such as oleic acid, and variations in their content can directly affect the spectral response intensity in this region. Therefore, under different blending ratios, the peak near 1656 cm^−1^ usually exhibits relatively pronounced intensity changes and shows high sensitivity to olive oil content.

Table 1 lists the vibrational assignments for characteristic peaks. The bands located at 1441 cm^−1^, corresponding to –CH_2_ bending vibration; 1265 cm^−1^, corresponding to =C–H scissoring vibration; and 1156 cm^−1^, corresponding to C–C vibration also exhibit certain degrees of variation. These peaks mainly reflect differences in fatty-chain structure and the degree of saturation or unsaturation, thereby providing auxiliary discriminative information for the model. However, in comparison, the variation amplitudes of these bands and their sensitivity to blending ratios are weaker than those of the 1656 cm^−1^ band.

### 3.2. Performance Comparison of Single-Task Learning Models

To evaluate the performance of single-task learning methods in detecting olive oil adulteration, this study constructed a classification model for adulteration type and a regression model for olive oil content separately, based on preprocessed Raman spectroscopy data, and compared several representative single-task approaches. Specifically, traditional machine learning models included support vector machine (SVM), random forest (RF), partial least squares (PLS), and extreme gradient boosting (XGBoost), where the first four were combined with two feature extraction strategies—principal component analysis (PCA) and linear discriminant analysis (LDA)—to construct the corresponding models [30]. In addition, one-dimensional convolutional neural network (1D-CNN) and long short-term memory (LSTM) networks were introduced as deep learning single-task baselines to examine the applicability of end-to-end feature learning in this context [31]. The classification and regression results of each model are presented in Table 2.

From an overall perspective, the different models exhibited pronounced differences in performance across the classification and regression tasks. For traditional machine learning models, those built with PCA-based features consistently outperformed those based on LDA features. Taking XGBoost as an example, it achieved a classification accuracy of 95.72%, a mean absolute error (MAE) of 2.96, and an R^2^ of 0.97 under PCA features, demonstrating the best overall performance. In contrast, under LDA features, although certain models still maintained high classification accuracy—SVM, for instance, attained 98.30% classification accuracy—their regression performance declined markedly, with an R^2^ of only 0.71. This indicates that LDA tends to enhance class separability while insufficiently preserving the continuous information required to characterize variations in adulteration ratio [12]. Similar trends were observed for RF, PLS, and XGBoost, suggesting that PCA is more suitable for simultaneously supporting both classification and quantitative analysis in the detection of edible oil adulteration.

From a model-level perspective, ensemble learning models exhibited strong competitiveness. Both RF and XGBoost achieved favorable results under PCA features, with RF attaining a classification accuracy of 93.10% and an R^2^ of 0.96, while XGBoost further improved to 95.72% and 0.97, highlighting its advantage in handling nonlinear relationships within Raman spectra. In comparison, PLS, a classical chemometric method with strong interpretability in spectral quantitative analysis, showed relatively weaker overall performance in this study. In particular, both its classification accuracy and regression performance decreased considerably under LDA features, indicating limited capacity to characterize this complex adulteration system [32]. SVM performed relatively well in the classification task, especially achieving high accuracy under LDA features, but its regression performance was less stable, reflecting that this method is more suited to categorical discrimination than to continuous content prediction.

With regard to deep learning single-task models, both 1D-CNN and LSTM directly took the preprocessed spectral sequences as input, thereby circumventing manual feature extraction. The results showed that 1D-CNN achieved a classification accuracy of 94.98%, an MAE of 5.75, and an R^2^ of 0.92. Although its overall performance surpassed that of some traditional models, it did not reach the optimal level. LSTM demonstrated stronger modeling capability, achieving a classification accuracy of 98.56%, an MAE of 1.88, and an R^2^ of 0.99, markedly outperforming 1D-CNN and most traditional machine learning models. This indicates that, compared with local convolutional feature extraction, LSTM more thoroughly captures long-range dependencies and global variation trends in the spectral sequences, rendering it more suitable for the task investigated in this study [33].

Although single-task models can attain high accuracy in either classification or regression tasks, they inherently require two independent models, making it difficult to jointly exploit the potential shared information between adulteration type identification and content prediction within a unified framework. As shown in Table 2, the proposed method achieved an overall optimal result under a single framework, with a classification accuracy of 99.15%, an MAE of 1.40, and an R^2^ of 0.99, outperforming all single-task baselines. These results demonstrate that multi-task collaborative modeling can further reduce quantitative prediction errors while maintaining high classification performance, thereby improving the consistency and practical utility of olive oil adulteration detection.

### 3.3. Comparison of Multi-Task Learning Models

Given that Raman spectra in the same spectral region concurrently carry information for category discrimination and content variation, adopting a multi-task learning framework to jointly model the two types of tasks is of considerable significance [34]. Accordingly, this section presents a comparative analysis of several typical multi-task learning architectures under a unified experimental setting, in order to evaluate the effectiveness of different strategies in handling classification and regression tasks.

**Figure 4:**
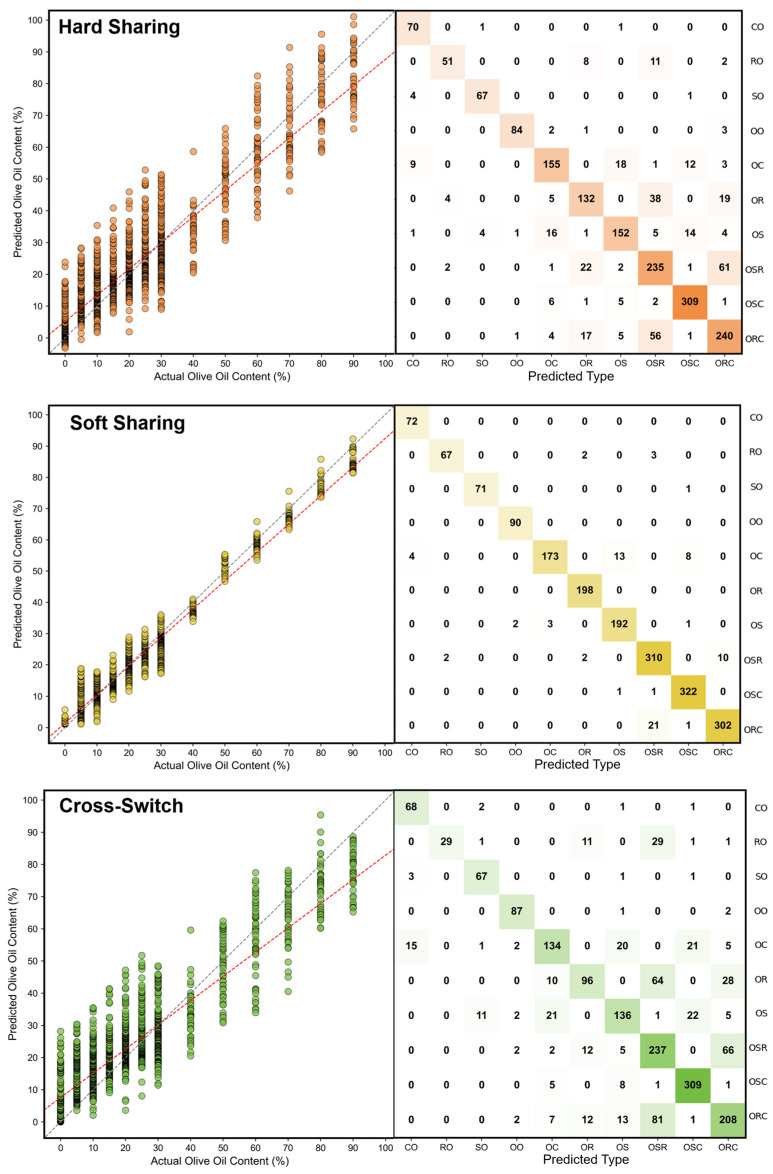

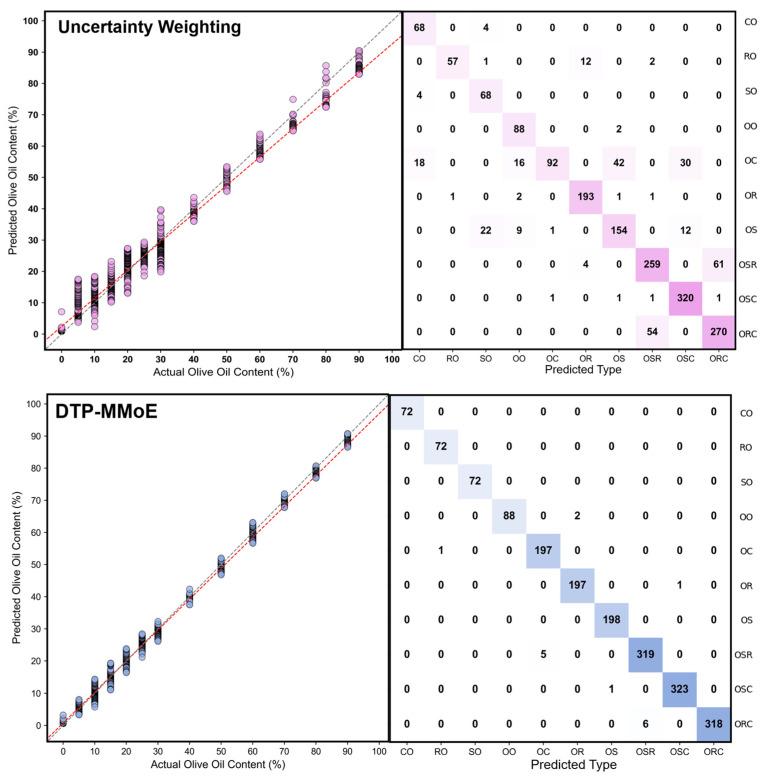
Confusion matrices and scatter plots of multi-task learning across different frameworks.

Table 3 summarizes the performance metrics of the different MTL architectures, and the overall results reveal the critical influence of model architecture design on task coordination. As can be seen from the table, pronounced performance differences across classification and regression tasks were observed among the different strategies. Figure 4 displays the confusion matrices and scatter plots of the multi-task models.

The Hard Sharing model, as the most fundamental multi-task learning structure, achieved a classification accuracy of 79.86% and a regression R^2^ of 0.840, yielding relatively low overall performance. This result indicates that forcing all tasks to share the same set of underlying features leads to notable task interference [35]. Because classification emphasizes learning discriminative boundaries, while regression requires characterizing continuous numerical relationships, the optimization directions in the feature space differ between the two; simple hard sharing cannot readily accommodate both requirements, thereby constraining model performance.

The Soft Sharing model maintains relatively independent parameter spaces for different tasks and enforces consistency among parameters through regularization constraints, thus alleviating task conflicts to a certain extent [6]. The experimental results show that this model performed favorably on the regression task (R^2^ = 0.973) while also achieving a classification accuracy of 95.99%, suggesting that, under moderately decoupled conditions, a multi-task model can better capture both the continuous variation information and the categorical discriminative features in the spectral data.

Although the Cross-Stitch network enables feature interaction between tasks through learnable linear combinations, its classification accuracy in this study was only 73.23%, representing a marked decline. This phenomenon indicates that, when substantial differences exist between tasks, unconstrained feature interactions may weaken discriminative features, thereby impairing classification performance [36]. This further suggests that multi-task learning requires not only information sharing but also effective control over the sharing process.

The Uncertainty Weighting model dynamically weights the losses of different tasks by introducing task-dependent uncertainty parameters, thereby achieving a degree of task balancing during training [32]. This method yielded relatively stable performance in the present experiment (classification accuracy = 83.81%, regression R^2^ = 0.853), outperforming the Hard Sharing model but still falling behind the Soft Sharing model overall. This indicates that adjusting weights solely at the loss level is insufficient to fully resolve task conflicts at the feature level.

Compared with the above methods, the proposed DTP-MMoE model achieves optimal performance across all metrics, with a classification accuracy of 99.15%, a regression R^2^ of 0.99, and significantly reduced mean absolute error. The performance improvement can be attributed to two aspects. On the one hand, the MMoE structure introduces multiple expert networks and employs task-specific gating mechanisms to produce weighted combinations of expert outputs, allowing different tasks to adaptively select more suitable feature representations. This achieves “selective sharing” at the feature level and effectively alleviates task conflicts. On the other hand, the DTP mechanism dynamically adjusts task-specific loss weights, enabling the model to adaptively allocate optimization priorities according to task difficulty during training, thereby balancing multiple tasks at the optimization level.

The performance of a multi-task learning model depends not only on whether parameters are shared, but also on the flexibility of the sharing strategy and the rationality of task weight allocation. By introducing a gating selection mechanism at the feature level and a dynamic weight adjustment mechanism at the loss level, the proposed DTP-MMoE model realizes effective collaborative optimization of classification and regression tasks, demonstrating significant advantages in olive oil adulteration detection.

### 3.4. Ablation Experiments

To further analyze the contribution of each key module in the DTP-MMoE model to the overall performance and to verify the rationality of its design, this section systematically evaluates the core components of the model through ablation experiments. Specifically, multiple model variants were constructed by varying three key factors—the number of expert networks, the gating mechanism, and the dynamic task prioritization (DTP) mechanism—and comparative analyses were conducted on the same dataset under identical experimental settings. The results are presented in Table 4.

Regarding the role of the gating mechanism, a comparison between Experimental Group 2 (the full model) and Experimental Group 4 (with the gating network removed) reveals that, in the absence of the gating mechanism, the model performance deteriorated substantially, with classification accuracy dropping to 77.99% and the regression R^2^ decreasing to 0.86. This result indicates that when all tasks share the same feature representations without task-specific selection capability, conflicts between the classification and regression tasks in the feature space are difficult to avoid, leading to an overall decline in performance. By assigning different expert weights to different tasks, the gating mechanism enables adaptive screening of shared features, allowing the model to dynamically select the optimal feature combination according to task requirements. It therefore constitutes one of the key factors contributing to the model’s enhanced performance.

With respect to the role of the dynamic task prioritization mechanism, a comparison between Experimental Group 2 and Experimental Group 3 (which replaced DTP with fixed weights) shows that, after removing the DTP mechanism, the classification accuracy decreased to 91.66% and the regression R^2^ dropped to 0.88. This phenomenon suggests that, in multi-task learning, fixed loss weights find it difficult to accommodate the varying learning difficulties of different tasks across training stages, easily causing one task to receive excessive attention or to be neglected during optimization. By dynamically adjusting the weights according to the relative changes in task losses, the DTP mechanism enables the model to adaptively balance the optimization processes of the classification and regression tasks during training, thereby improving the overall performance.

Concerning the influence of the number of expert networks, a comparison between Experimental Group 1 (two experts) and Experimental Group 2 (four experts) indicates that increasing the number of experts significantly improved model performance, suggesting that a richer expert structure facilitates the capture of feature patterns at different levels within the spectral data. However, when the number of experts was further increased to eight (Experimental Group 5), a declining trend in performance was observed (classification accuracy = 94.87%, regression R^2^ = 0.96), indicating that an excessive number of experts may introduce redundant features, increase model complexity, and pose a risk of overfitting. Therefore, the number of experts needs to strike a balance between feature representation capacity and model generalization ability.

The performance improvement of the DTP-MMoE model arises from the synergistic effects of multiple key modules. The gating mechanism achieves task-specific “selective sharing” at the feature level, effectively mitigating feature conflicts among multiple tasks; the DTP mechanism enables dynamic adjustment of task weights at the optimization level, ensuring balanced optimization throughout the training process; and an appropriate number of expert networks provides the model with sufficient, but not excessive, feature representation capacity. The combined action of these three components allows the model to achieve efficient synergy between classification and regression tasks, thereby outperforming other comparative methods.

### 3.5. Training Process Analysis

To further analyze the role of the DTP mechanism during multi-task training, this section presents a visual analysis of the loss variations in the classification and regression tasks throughout the training process. Because the classification task uses cross-entropy loss whereas the regression task uses mean absolute error (MAE), the two losses differ markedly in numerical magnitude (classification loss ≈ 0.1–0.3; regression loss ≈ 500–1500). If fixed weights were adopted for direct summation, the total loss would be dominated by the regression task with its substantially larger magnitude, causing the classification task to be easily neglected during optimization and thereby leading to imbalanced training. Although conventional methods can achieve simultaneous decreases in both task losses through fixed weighting or manual hyperparameter tuning, they are incapable of adaptively allocating optimization resources. As a result, the low-magnitude task may be overshadowed during early training stages and converge slowly, while fixed weights cannot accommodate the varying difficulty of tasks across different phases, leading to imbalanced convergence.

**Figure 5:**
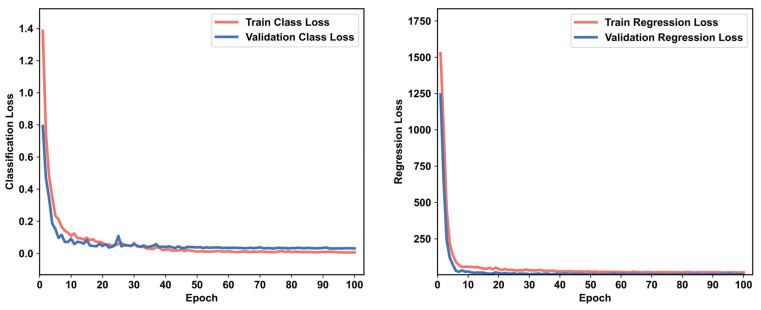
Training loss curves of the DTP-MMoE model for classification and regression.

Figure 5 displays the loss curves and validation curves for the classification and regression tasks during training. Under the action of the DTP mechanism, the optimization intensity for each task is automatically adjusted based on the current changes in task loss, such that tasks that converge more slowly or exhibit higher losses are prioritized for gradient updates. When the classification task loss is small, the DTP mechanism still ensures that it receives sufficient optimization attention, preventing it from being overshadowed by the high-magnitude task.

### 3.6. Task Correlation Analysis and Basis for Multi-Task Modeling

To further investigate the feature utilization mechanism of the DTP-MMoE model in the spectral space and to elucidate the intrinsic basis for jointly modeling adulteration type identification and content prediction, this section analyzes the contribution of different Raman shifts to the model’s decision-making by combining feature importance analysis derived from expert network outputs with gating weight distribution across tasks.

#### 3.6.1. Feature Importance Analysis of Salient Spectral Bands

**Figure 6:**
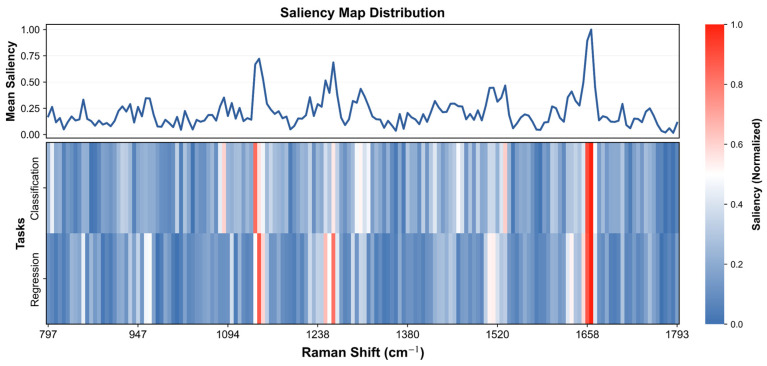
Heatmap of feature importance for the two tasks in the DTP-MMoE.

Figure 6 presents the feature importance heatmap across the 800–1800 cm^−1^ spectral region. As shown, the model’s responses to different Raman shifts exhibit a markedly non-uniform distribution, with several spectral bands demonstrating notably enhanced response signals. Notably, around approximately 1658 cm^−1^, all expert networks show relatively high importance responses. In the normalized heatmap, this region exhibits good consistency across different experts, indicating that this band makes a stable and significant contribution to the model’s decision-making process for both tasks.

The average feature importance curve further corroborates the above observation, with a globally maximum peak appearing in the approximately 1660–1670 cm^−1^ range, suggesting that this band occupies a dominant position in the overall feature space. This region corresponds to the stretching vibration of C=C bonds in unsaturated fatty acids [22]. Olive oil is particularly rich in monounsaturated fatty acids such as oleic acid; therefore, as the adulteration ratio increases, the relative content of such unsaturated fatty acids decreases, leading to a corresponding decline in spectral intensity in this region. Consequently, this band exhibits high sensitivity to variations in adulteration ratio for the regression task while also providing discriminative information for classifying different adulteration types. The model’s strong and consistent attention to this region across both classification and regression tasks confirms that it serves as a shared information hub, providing a solid chemical basis for multi-task collaborative modeling.

In addition to the 1658 cm^−1^ band, a relatively pronounced response enhancement is also observed around approximately 1106 cm^−1^. This band is primarily associated with C–C bond vibrations related to the fatty chain structure and provides auxiliary structural information. Furthermore, localized enhancement regions can be observed near 1251 cm^−1^, 1393 cm^−1^, and 1532 cm^−1^, although their responses vary across experts, exhibiting a relatively strong degree of inconsistency. This phenomenon suggests that while these bands contain useful information, their contributions are more task- or expert-dependent rather than universally shared.

#### 3.6.2. Expert Specialization in Spectral Feature Attention

**Figure 7:**
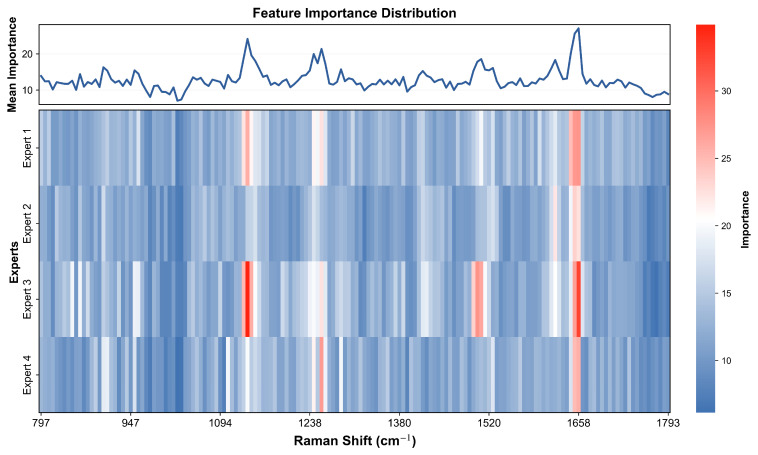
Heatmap of feature importance for the four expert networks in the DTP-MMoE.

With respect to inter-expert differences, the responses of different expert networks show notable divergence in certain spectral bands, particularly in regions of moderate importance (e.g., the 1200–1500 cm^−1^ range), where the experts display a certain degree of dispersion in their feature attention. Figure 7 presents the feature importance heatmap of the four expert networks individually, providing a more granular view of expert-level spectral attention. It can be observed that Expert 1 and Expert 2 exhibit relatively high response intensity around the 1658 cm^−1^ band, which is consistent with their dominant role in the regression task (as shown later in the gating weight analysis). In contrast, Expert 3 and Expert 4 show more distributed attention across multiple bands, including 1106, 1251, and 1441 cm^−1^, which aligns with their greater contribution to the classification task. This complementary attention pattern across experts indicates that the model achieves an implicit feature specialization mechanism through the MMoE structure: some experts focus more on capturing key bands closely related to adulteration ratio variations (e.g., the 1669 cm^−1^ band), while others comprehensively model multiple characteristic peaks, thereby forming complementary representations within the shared feature space.

The feature importance distribution of the model aligns well with the known chemical characteristic peaks in Raman spectra, and the prominent response around 1658 cm^−1^, in particular, further validates the important role of this band in olive oil adulteration detection. Simultaneously, the differential responses of different experts in the feature space, as visualized in Figure 6 and Figure 7, indicate that the DTP-MMoE model can achieve an effective balance between sharing and specialization, thereby enhancing the overall performance of multi-task learning.

#### 3.6.3. Task Decoupling via Gating-Based Expert Selection

Adulteration type identification constitutes a discrete classification task, which focuses more on the categorical boundaries and discriminative features among different oil samples, whereas olive oil content prediction constitutes a continuous regression task, which places greater emphasis on the continuity and sensitivity of the spectral response as a function of concentration. Because these two types of tasks differ in their optimization objectives and feature requirements, adopting a simple sharing strategy often results in mutual interference between tasks, limiting performance improvement. This explains why the Hard Sharing model in Section 3.3 exhibited relatively poor performance, whereas the DTP-MMoE model, through its “multi-expert + gating selection” mechanism, achieved effective task decoupling.

**Figure 8:**
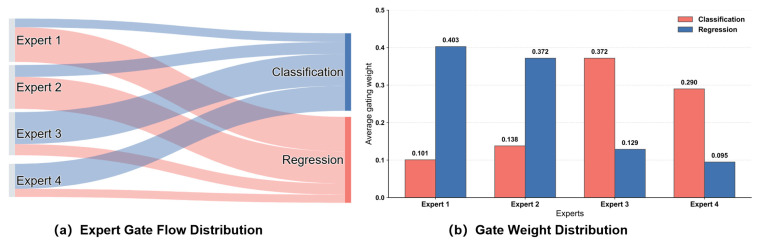
Average gating weights of different experts for the classification and regression tasks.

Figure 8 illustrates the distribution of the average gating weights assigned by the classification and regression tasks to the four expert networks. It can be observed that the classification task primarily relies on Expert 3 and Expert 4, with Expert 3 receiving the highest weight, whereas the regression task mainly depends on Expert 1 and Expert 2, with Expert 1 playing a dominant role. This indicates that the invocation patterns across the shared expert pool differ markedly between tasks. The model does not adopt a uniform feature representation for all tasks; instead, it enables task-specific scheduling of the expert networks through the gating mechanism [37].

From a feature decoupling perspective, the introduction of the gating mechanism allows the model to maintain partially shared representations while avoiding direct interference between different tasks. Compared with the traditional hard parameter sharing model, DTP-MMoE, through its “multi-expert + gating selection” architecture, enables different tasks to dynamically select the most suitable feature combinations from the shared feature repository, thereby achieving “soft decoupling” at the feature level [38]. This mechanism not only improves feature utilization efficiency but also provides a more flexible means of representation for synergistic optimization across multiple tasks [39].

The differential distribution of gating weights across tasks also indicates that a certain degree of expert specialization has emerged within the model. Some experts (e.g., Expert 1 and Expert 2) are primarily responsible for extracting key features associated with concentration variations, whereas others (e.g., Expert 3 and Expert 4) focus more on capturing the overall spectral profile structure or auxiliary discriminative information. Such implicit functional differentiation is consistent with the original design intent of the MMoE model and contributes to enhancing the model’s expressive capacity and generalization performance in multi-task scenarios [40]. This specialization pattern also explains why increasing the number of experts from two to four improved model performance (as shown in Section 3.4, Experimental Groups 1 vs. 2), while further increasing to eight experts led to performance degradation due to over-specialization and redundancy.

### 3.7. Generalization Experiments

To evaluate the generalization ability and practical applicability of the proposed model, commercially available blended oil (BO) samples that were not involved in model training or internal validation were selected for an external validation experiment. All samples had a nominal olive oil content of 5%, representing commercial products with complex compositions and inherent uncertainties typical of real market conditions. The model was directly applied to this dataset without retraining or parameter adjustment, thereby simulating the prediction performance under real deployment scenarios.

**Figure 9:**
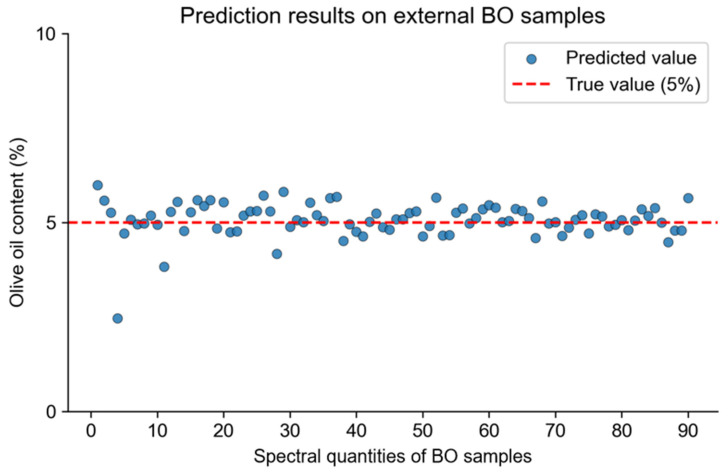
Scatter plot of BO predictions using a DTP-MMoE-based multi-task learning model.

Figure 9 shows the prediction results of the BO samples. On the BO dataset, the model yielded a mean predicted value of 5.069%, indicating a slight overall overestimation but with minimal error. The MAE was 0.317%, the RMSE was 0.459%, the MAPE was 6.34%, and the median absolute error was 0.256%, all remaining at low levels, suggesting that the model achieved high prediction accuracy on the external data. Further analysis showed that the 95% confidence interval for the predicted mean was [4.974%, 5.165%] and that for the bias was [−0.026%, 0.165%]. The fact that the bias interval included zero indicates the absence of significant systematic error, demonstrating good statistical stability in the overall predictions.

The model’s performance on the external commercial samples showed only a slight decline relative to the internal validation, indicating good adaptability to shifts in data distribution. This result suggests that the model based on DTP-MMoE does not merely memorize the training data but can effectively capture the intrinsic relationship between spectral features and olive oil content, thereby possessing a certain degree of generalizability. Moreover, the model’s ability to accurately predict samples with a low olive oil content (5%) demonstrates its high sensitivity in adulteration detection, highlighting its potential practical application value.

## 4. Conclusions

This study proposes a novel method based on a Dynamic Task Prioritization Multi-Gate Mixture-of-Experts (DTP-MMoE) model, which leverages Raman spectroscopy to simultaneously achieve the qualitative identification of olive oil adulteration types and quantitative prediction of adulteration ratios. The experimental results demonstrate the excellent performance of the model, with a classification accuracy of 99.15% and a coefficient of determination (R^2^) for regression prediction reaching as high as 0.9903. This superiority is primarily attributed to the decoupling mechanism for spectral features and the dynamic balancing of loss gradients between the classification and regression tasks enabled by the DTP mechanism, which effectively resolves feature conflicts between heterogeneous tasks and markedly enhances the model’s generalization ability. Compared with conventional chemometric methods that rely on manual preprocessing, the DTP-MMoE model realizes end-to-end automated processing of raw spectra, eliminating manual intervention and substantially improving detection efficiency. This study provides an efficient, non-destructive detection scheme for olive oil authenticity verification, and future work will further explore its application potential in the authenticity verification of other high-value food products.

## Figures and Tables

**Table 1:** Raman characteristic peaks of edible oils in the 1000–1800 cm^−1^ spectral region.

Raman Shift/cm^−1^	Chemical Bond	Vibrational Mode	References
1156	β-carorene	C–C bending vibration	[20,21]
1265	RHC=CHR	=C–H scissoring vibration	[22,23]
1300	-CH_2_	C–H twisting vibration	[24,25]
1441	-CH_2_	C–H bending vibration	[26,27]
1526	β-carorene	C=C bending vibration	[20,21]
1658	RHC=CHR	C=C stretching vibration	[28,29]

**Table 2:** Performance comparison of single-task learning.

Models	Feature Selection	Classification	Regression	
		Accuracy (%)	MAE	R^2^
SVM	PCA	95.35	5.26	0.93
	LDA	98.3	11.08	0.71
RF	PCA	93.1	3.49	0.96
	LDA	97.86	9.37	0.77
PLS	PCA	83.84	8.65	0.84
	LDA	80.68	13.86	0.58
XGBOOST	PCA	95.72	2.96	0.97
	LDA	97.38	9.17	0.78
1D-CNN	/	94.98	5.75	0.92
LSTM	/	98.56	1.88	0.99
Ours	/	99.15	1.4	0.99

**Table 3:** Performance comparison of multi-task learning across different frameworks.

Model	Accuracy (%)	MAE	R^2^	F1-Score	Precision	RPD	RER	Running Time (s)/Epoch
Hard Sharing	79.86 ± 5.03	7.68 ± 2.15	0.840 ± 0.022	0.910 ± 0.034	0.919 ± 0.028	2.57 ± 0.22	8.62 ± 0.41	0.61
Soft Sharing	95.99 ± 1.02	3.47 ± 0.55	0.973 ± 0.006	0.960 ± 0.012	0.965 ± 0.009	5.94 ± 0.11	19.71 ± 0.16	0.53
Cross-Switch	73.24 ± 7.56	8.41 ± 1.82	0.821 ± 0.013	0.727 ± 0.045	0.742 ± 0.041	2.37 ± 0.23	7.93 ± 0.43	0.63
Uncertainty Weighting	83.81 ± 4.37	7.44 ± 2.14	0.853 ± 0.015	0.8418 ± 0.046	0.861 ± 0.039	2.76 ± 0.20	9.10 ± 0.39	0.62
DTP-MMoE	99.15 ± 0.31	1.40 ± 0.13	0.990 ± 0.003	0.985 ± 0.005	0.985 ± 0.004	12.85 ± 0.04	43.23 ± 0.13	0.74

**Table 4:** Comparison of ablation experiment results.

Experimental Group	Number of Experts	Gating Network	DTP Weighting	Accuracy	MAE	R^2^	RPD	RER
1	2	√	√	92.84	6.01	0.91	3.32	10.99
2	4	√	√	99.15	1.4	0.99	12.85	43.23
3	4	√	×	91.66	7.35	0.88	2.89	9.48
4	4	×	√	77.99	7.89	0.86	2.67	8.94
5	8	√	√	94.87	2.61	0.96	5.03	16.85

## Data Availability

The original contributions presented in the study are included in the article/Supplementary Materials; further inquiries can be directed to the corresponding author.

## Supplementary Materials

The following supporting information can be downloaded at https://www.mdpi.com/article/10.3390/foods15112030/s1: Figure S1: Total training loss curves based on DTP-MMoE; Table S1: The composition of ternary blended olive oils; Table S2: The configuration of training environment.
