# Supplementary material for: A Multi-Task Learning Model Based on DTP-MMoE for Identification of Olive Oil Multi-Adulteration Using Raman Spectroscopy
Source: Foods. 2026 Jun 5;15(11):2030. doi: 10.3390/foods15112030 (PMC13256638; doi:10.3390/foods15112030)
Supplement: Supplementary file 1 [file foods-15-02030-s001.zip › foods-4319596-supplementary.pdf]

A Multi-Task Learning Model Based on DTP-MMoE for Identification of Olive Oil Multi-Adulteration Using Raman Spectroscopy

Xuewen Qin [1,†], Yulong Chen [2,†], Bing Li [1,*], Shan Zeng [1], Gaoxiang Mei [1] and Chen Yu [1]

[1] School of Mathematics and Computer Science, Wuhan Polytechnic University, Wuhan 430023, China
[2] College of Medicine and Health Science, Wuhan Polytechnic University, Wuhan 430023, China
[*] Correspondence: binglee@whpu.edu.cn; Tel.: +86-027-85504742
[†] These authors contributed equally to this work.

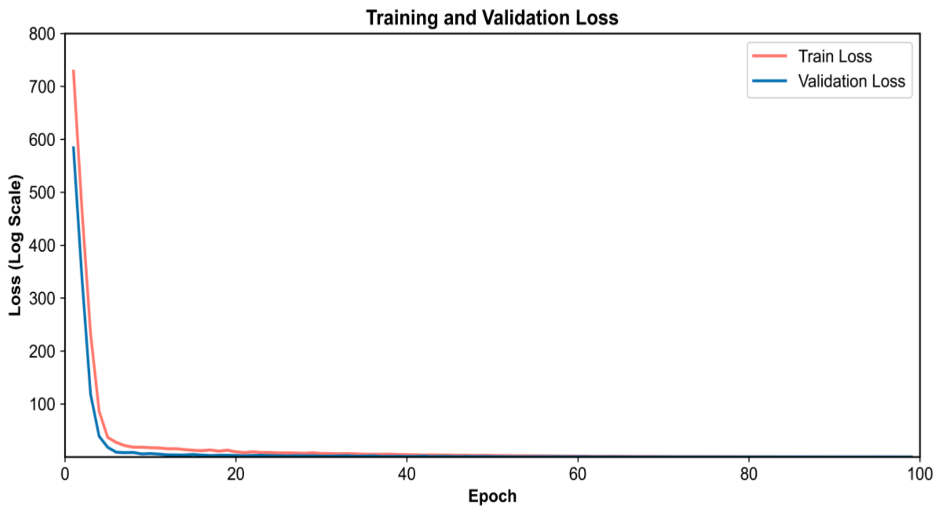

Figure S1. Total training loss curves based on DTP-MMoE.

Table S1. The composition of ternary blended olive oils.

**OSC**	OO (%)	SO (%)	CO (%)	**OSR**	OO (%)	SO (%)	RO (%)	**ORC**	OO (%)	RO (%)	CO (%)
OSC-1	5	25	70	OSR-1	5	25	70	ORC-1	5	25	70
OSC-2	5	45	50	OSR-2	5	45	50	ORC-2	5	45	50
OSC-3	5	70	25	OSR-3	5	70	25	ORC-3	5	70	25
OSC-4	10	20	70	OSR-4	10	20	70	ORC-4	10	20	70
OSC-5	10	45	45	OSR-5	10	45	45	ORC-5	10	45	45
OSC-6	10	70	20	OSR-6	10	70	20	ORC-6	10	70	20
OSC-7	15	20	65	OSR-7	15	20	65	ORC-7	15	20	65
OSC-8	15	40	45	OSR-8	15	40	45	ORC-8	15	40	45
OSC-9	15	65	20	OSR-9	15	65	20	ORC-9	15	65	20
OSC-10	20	20	60	OSR-10	20	20	60	ORC-10	20	20	60
OSC-11	20	40	40	OSR-11	20	40	40	ORC-11	20	40	40
OSC-12	20	60	20	OSR-12	20	60	20	ORC-12	20	60	20
OSC-13	25	20	55	OSR-13	25	20	55	ORC-13	25	20	55
OSC-14	25	35	40	OSR-14	25	35	40	ORC-14	25	35	40
OSC-15	25	55	20	OSR-15	25	55	20	ORC-15	25	55	20
OSC-16	30	20	50	OSR-16	30	20	50	ORC-16	30	20	50
OSC-17	30	35	35	OSR-17	30	35	35	ORC-17	30	35	35
OSC-18	30	50	20	OSR-18	30	50	20	ORC-18	30	50	20

Table S2. The configuration of training environment.

**Category**	**Details**
Python Version	3.11.7
Deep Learning Framework	PyTorch
Data Processing Libraries	NumPy, Pandas, scikit-learn
Hardware	NVIDIA GeForce RTX 4090 D with CUDA acceleration
Operating System	Windows 11
